# Hirschsprung disease in an adult with intestinal malrotation and volvulus: an exceptional association

**DOI:** 10.1186/s13256-019-2020-0

**Published:** 2019-04-29

**Authors:** Elise Lupon, François Labbe, Emile Nini, Sixte Sondji

**Affiliations:** 1 Department of Plastic Surgery, University Toulouse III Paul Sabatier, 1 Avenue du Pr jean Poulhes, 31400 Toulouse, France; 2 Department of Visceral Surgery, Carcassonne Hospital, 1060 Chemin de la Madeleine, 111000 Carcassonne, France

**Keywords:** Adult Hirschsprung disease, Malrotation intestinal, Small bowel volvulus, Constipation, Multiple occlusive syndrome, Chronic occlusion

## Abstract

**Background:**

Hirschsprung disease is a neonatal discovery in almost all cases, and the association of Hirschsprung disease in adults with symptomatic intestinal malrotation is unusual. This combination delays diagnosis and can lead to mistake in surgical strategy.

**Case presentation:**

A 43-year-old patient with a history of colectomy for colonic inertia and megadolichocolon was admitted to the Carcassonne Hospital emergency room for a volvulus of small bowel obstruction in a chronic intestinal obstruction context with episodes of acute, variable-looking occlusive syndromes. Intestinal malrotation was discovered during surgical small bowel detorsion. The acute occlusion syndrome recurred after the procedure. In view of the unfavorable evolution, an emptying of the dilated small bowel and a discharge ileostomy upstream of the rectum were performed. In the face of postoperative improvement, rectal manometry and deep full parietal rectal biopsies made it possible to highlight the diagnosis of Hirschsprung disease. The patient thus had functional acute occlusive syndromes and chronic occlusion due to Hirschsprung disease of attenuated form and acute organic occlusive syndromes related to her incomplete common mesentery.

**Conclusions:**

This rare association, which may be responsible for delayed diagnostic and therapeutic wandering, highlights the importance of performing manometry and deep full parietal biopsies before a colectomy for colonic inertia, as well as the possibility of suggesting a common Hirschsprung disease and/or mesentery in an adult with multiple occlusive syndromes of variable appearance.

## Introduction

Hirschsprung disease (HD) usually results in occlusive episodes in the neonatal period. The diagnosis of HD in adulthood is uncommon and is reported to correspond to 2% of adult patients with chronic, rebellious constipation. The symptomatology of these patients is refractory constipation and abdominal pain of the colic type [1, 2].

Intestinal malrotations are most often revealed in the neonatal period by acute symptomatology with volvulus frequently affecting the small bowel throughout the territory of the superior mesenteric artery. The association of HD and a malrotation is very rare. It alters the clinical and radiological presentation, complicates diagnosis, and may delay treatment of either condition [3, 4]. We report the unusual observation of an adult patient with multiple episodes of acute occlusions of variable profiles in a chronic intestinal obstruction field revealing a previously unknown HD associated with intestinal malrotation.

## Case presentation

A 43-year-old patient was referred to the Carcassonne Hospital emergency room for acute occlusive syndrome. This married patient, housewife, and mother of six children, with a strong religious education, did not smoke or drink alcohol. There was no significant digestive history in her family. Her obstetrical and gynecological history was marked by six pregnancies, including three caesarean sections and a tubal ligation. She also had a heavy digestive history marked by several episodes of acute functional occlusive syndromes, severe chronic constipation, and chronic abdominal pain. In 2007, the patient had a subtotal colectomy with ileorectal anastomosis for a diagnosis of colonic inertia in a megadolichocolon context. The anatomopathology of the colon upon examination was normal. The chronic occlusive bowel disorder had persisted postoperatively, and the patient then had numerous acute functional occlusive episodes that either spontaneously resolved or occurred after colo-exsufflation of the small bowel (allowed by colectomy). These occlusive episodes were aggravated by pregnancy. In 2012, the patient had a small intestinal volvulus treated with surgical detorsion, but no specific cause of volvulus was mentioned in the operating report. The low endoscopy performed at the end of the course made it possible to assess the integrity of the anastomosis, which appeared to be healthy, as well as to perform superficial biopsies, which showed acute nonspecific ileitis and chronic nonspecific rectitis. In 2016, following several other episodes of acute obstructive syndrome, new biopsies were performed, which revealed no small bowel lesions and nonsuspicious nonspecific inflammatory changes in ileocolic anastomosis.

**Fig. 1:**
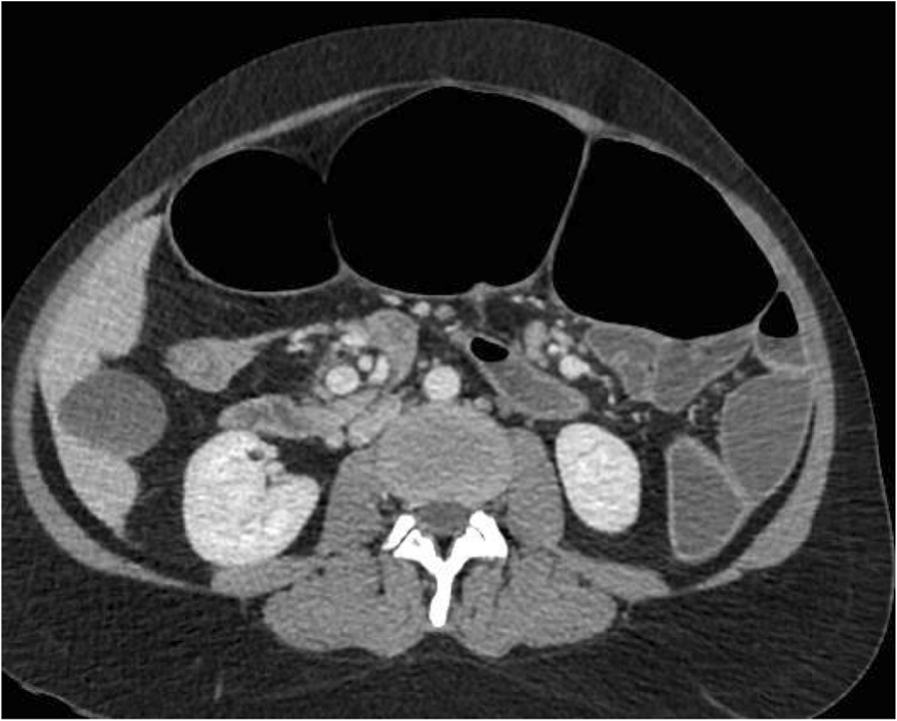
Abdominal axial cross-sectional computed tomography showing hydroaeric small intestine distention with pneumatosis of the small intestine loops

**Fig. 2:**
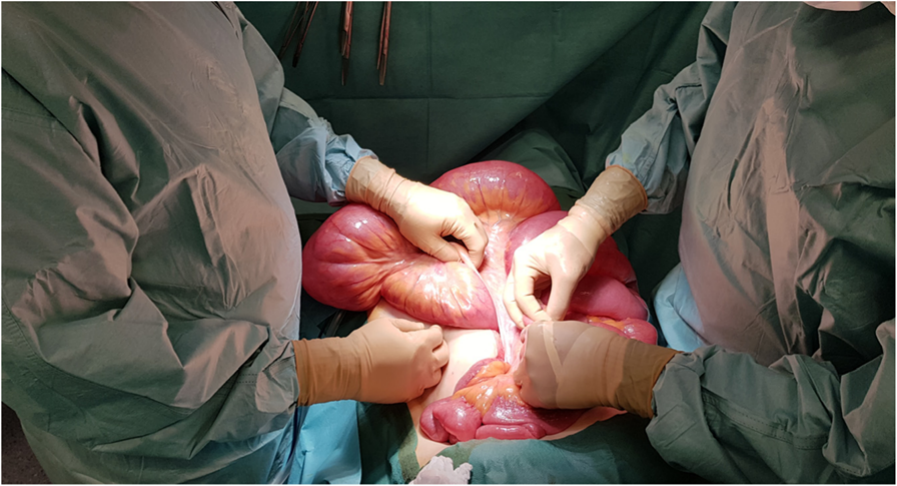
Volvulus of the small intestine with torsion of the mesentery and major dilation of the small intestine

**Fig. 3:**
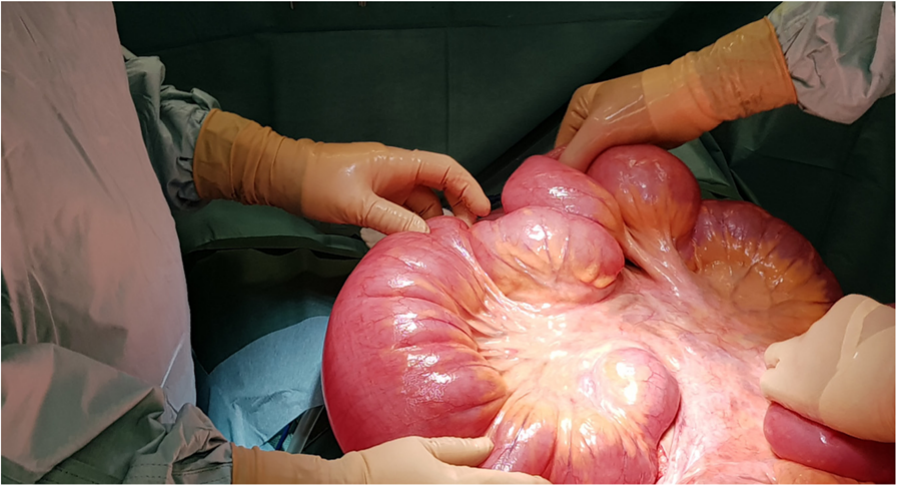
Highlighting of a mesentery detached from the posterior wall and very elongated after manual detorsion

The clinical picture was of acute intestinal obstruction with hemodynamic failure. Indeed, the patient was tachycardic at 148 beats/min and hypotensive with 75 mmHg systolic pressure, and she had marbling of the lower limbs. Her temperature was 37.9 °C. Computed tomography showed major hydroaeric distention and parietal pneumatosis of the small intestine loops (Fig. 1). The patient was taken to the operating room emergently. The surgical procedure performed through median laparotomy revealed an ileal volvulus on a mesentery largely detached from the posterior wall and very elongated, suggesting an incomplete common mesentery of atypical appearance, given the history of colectomy (Fig. 2). No adhesions were found. The very dilated small intestine was manually detorsioned, and no small intestine resection was necessary (Fig. 3). An ileotomy with small intestinal emptying allowed recovery of the bowel’s size. The patient was treated with painkillers paracetamol and morphine (skenan) and antiemetics. A nasogastric tube was necessary in this occlusion context. Short-term parenteral nutrition was implemented. The patient received vascular filling with crystalloids. Oxygen therapy of 3 L/min was required for admission. The patient received preventive anticoagulation and gastric protectors throughout the hospitalization.

The patient’s postoperative course was unfavorable with no resumption of transit as well as severe pain resistant to morphine, and distention and a slight bloating marked the abdominal examination. The result of the neurological examination was normal. CT showed a recurrence of small intestine dilation. Following the failure of the colo-exsufflation attempts, the patient was taken back to the operating room on day 13 of her previous intervention by median laparotomy. During surgical exploration, no adhesions were found, and there was major dilation upstream of the ileorectal anastomosis. A 10-cm ileotomy was performed above the anastomosis, which allowed draining of the small intestine content and manual exploration of this anastomosis. So, we paid particular attention to this anastomosis, which seemed to be the cause of the patient’s clinical picture, given the level of dilation. This anastomosis appeared perfectly open and integrated into the palpation. A lateral ileostomy at the ileotomy was performed to support the hypothesis of an obstacle under the anastomosis at the rectum.

The postoperative evolution was favorable on the digestive level with a resumption of transit and an improvement in the patient’s general condition. However, she developed postoperative anemia, which was treated with iron sucrose. Manometry was performed, which showed the absence of a rectoanal inhibitory reflex, and full parietal deep biopsies confirmed a histological aspect compatible with HD, in particular with the absence of nerve nodes in the rectal parietal wall thickness.

Six months later, the patient was stable, and the stoma had improved the symptoms associated with chronic occlusion. She did not have a new episode of acute occlusion in the meantime. The patient must now consider, first, a surgical reoperation to treat her incomplete common mesentery, which is theoretically always at risk of volvulus. However, her wishes must be decided between the permanent wearing of her stoma, which she can bear very well, and finally a new surgical procedure to treat her illnesses without stoma. This would consist of resection of the rectum with ileoanal anastomosis allowing a restoration of her body integrity at the risk of chronic postoperative anal incontinence.

## Discussion

We have reported the surprising and long-unknown association of HD and intestinal malrotation in an adult woman, symptomatic of these two diseases. We share this case because it is unique; indeed, to the best of our knowledge, no such association of discovery in adulthood has previously been reported in the literature.

HD is expressed by a congenital absence of neuronal ganglion cells, derived from the neural crest, in the submucous membrane in the Meissner plexus and in the muscularis in the Auerbach plexus. Its diagnosis, usually neonatal, is confirmed in adults by manometry showing the absence of rectoanal inhibitory reflex. Deep biopsies are performed in case of diagnostic doubt and may show marked hyperplasia of the nerve threads [1, 5].

Unrecognized HD until adulthood represents 2% of patients with chronic, rebellious constipation and affects men more often (four men for every woman). The relatively good tolerance of the disease is mainly due to a short aganglionic zone, less than 10 cm long, and an upstream colon keeping enough propulsive force to counter distal obstruction [1]. In retrospect, HD could have been suspected on the basis of our patient’s history found during the interview (megadolichocolon, manual evacuation of stools, and evacuating enemas since childhood).

Once the diagnosis is well established in adults, the intervention is almost systematically indicated [1]. The evolution leads not only to worsening constipation by continuing low-noise distention of the upstream colon, as well as undernutrition and alteration of the general state, but also to sudden complications such as intestinal perforation, severe respiratory failure due to major abdominal distention [6], or necrotizing enterocolitis with sometimes fatal septic shock [7–9].

The surgical strategy for HD is based on the length of the achalasic zone, the length and reversibility of colonic dilation, the nutritional status of the patient, and the experience of the surgeon. The principle of transanal myectomy is to remove the spasm from the aganglionic zone when it is very short [10, 11]. The principle of sigmoid rectal resection with coloanal anastomosis and Swenson’s operation is to remove the aganglional part of the rectum and the irreversibly distended part of the upstream colon [12, 13]. The Duhamel technique is based on the principle of a short circuit of the aganglionic zone, lowering the healthy colon behind the diseased rectum left in place [14]. This technique was described at a time when rectal dissection was very difficult and morbid: The disadvantages of the operation were considered acceptable in terms of mortality and morbidity of rectal excision. The principle of Soave’s operation consists in resecting the distended colon and the upper part of the pathological rectum, but stopping dissection before approaching the lower rectum, for the same reasons as before [15]. The colon is lowered through the rectum, from which the mucosa has been removed. The surgical technical difficulty was related to the length of the remaining colon in our colectomized patient. Its treatment is currently being discussed by a specialized team.

Intestinal malrotations are congenital anomalies of rotation and fixation of the intestine and mesentery. During embryonic development, there is normally a rotation of the primary intestinal loop at 270 degrees. Early termination of this rotation is responsible for the complete (90 degrees) or incomplete (180 degrees) common mesentery. These pathologies are asymptomatic if transit is possible. The prevalence of common mesentery in adults is estimated at 0.2–0.5% of the population, and this pathology is the main risk factor for small intestine volvulus [16]. Small intestine volvulus is itself a rare disease in adults and is estimated to affect 1.7 in 100,000 persons per year in Western countries. This pathology is mainly of primary etiology on intestinal malrotation [17]. The Ladd procedure allows the incomplete common mesentery to be treated by repositioning it as a complete 90-degree common mesentery. This involves positioning the entire small intestine in the right hemiabdomen and the entire colon in the left hemiabdomen. Pexies (surgical fixations), which cause recurrences and increased morbidity due to the risk of internal hernias, release, and straps, must be avoided [18]. Because our patient has been colectomized, the conventional procedure is not possible, and repositioning the small intestine on the right alone may not be the most appropriate strategy for the left hemiabdominal emptiness she would have on occasion. The intervention necessary to treat the intestinal malrotation of this colectomized patient will be referred to a specialized center.

The colectomy was performed in a private hospital on the basis of diagnosis of colonic inertia without further investigation of constipation and volvulus. This pathology concerns less than 10% of patients with severe constipation resistant to medical treatment. Surgical treatment consisted of subtotal colectomy with ileorectal anastomosis, and the persistence of symptomatology was interpreted as a therapeutic failure, which is described in one in ten patients treated surgically for colonic inertia. This erroneous diagnosis could have been avoided by following the recommendations concerning the preoperative assessment in this context. Thus, the exhaustive colorectal assessment includes a colonoscopy (looking for an organic cause), a barium enema (looking for a megarectum), a measurement of colonic transit time, and a rectogram. The recommendations emphasize the essential nature of anorectal manometry before surgery, making it possible to eliminate HD [19].

The analysis of the colectomy operating room revealed that it was probably performed on a portion of a healthy colon. Indeed, HD is rectosigmoidal in 80% of cases [20], and the analysis of the upstream portion does not allow the diagnosis of HD. The first rectal biopsies performed in our patient before 2016 were superficial and did not allow the diagnosis of HD. However, the recommendations on how to carry out these biopsies are very precise and well codified. These should take the full parietal thickness and interest the posterior surface of the rectum, starting from the pectinate line over a height of 6 cm, and should be oriented. Biopsies should benefit from a conventional histological examination (using H&E) and an acetylcholinesterase study (showing an overexpression of this activity in HD). It is therefore not surprising that HD was not found on these biopsies.

The clinical picture of our patient was complex, involving several causes of occlusive syndromes of different presentations, and the variability of the centers in which the patient was managed contributed to the delayed diagnosis. On one hand, HD was responsible for acute functional obstructive episodes (by loss of peristalsis of the aganglionic zone and progressive dilation of the upstream segment) and chronic obstruction syndrome (by stasis upstream of the nonfunctional rectum); on the other hand, intestinal malrotation responsible for incomplete common mesentery was at the origin of episodes of organic obstructive syndromes expressed by the volvulus. The main differential diagnoses mentioned in front of this context were the presence of flanges or morphine intoxication with reflex ileus during the second intervention. It was during the operation, before the discovery of the dilation of the small intestine to the ileorectal anastomosis, integrated in addition, and the absence of obstacles or volvulus that HD was suspected. No flanges were ever found in our young, multioperated patient. The modified anatomy of the patient after the colectomy and the rarity of this pathology in adulthood probably caused the absence of treatment of this atypical mesentery, which had not been able to escape the previous actors in its surgical management.

The association of these pathologies is rare. HD is associated with other abnormalities in about 16% of cases (range, 5–30%): trisomy 21, genitourinary, skeletal, neurological, cardiac, and digestive abnormalities. It is estimated that 12% of associations of HD with other abnormalities are digestive, including intestinal malrotation in 0.3–1.4% of HD [4, 21]. The association of HD and malrotation is probably fortuitous. However, antenatal ischemia may be responsible for a failure to migrate neural crest-derived cells [4]. A reverse mechanism (volvulus and atresia secondary to HD) is not excluded [22]. Another hypothesis would be that the motor disorders induced by HD favor volvulus accidents on a common mesentery, which, in an isolated state, would have remained asymptomatic. Although a few pediatric cases have been reported [23–26], we found no adult cases of such an association in our review of the French and English literature.

## Conclusions

This anecdotal report highlights the importance of preoperative explorations, as well as respect for their modalities and interrogation in the surgical treatment of colic disorder, and it evokes the possibility of searching for a common HD and/or mesentery even in an adult.

## Abbreviation

HD: Hirschsprung disease
